# Skin lesion classification of dermoscopic images using machine learning and convolutional neural network

**DOI:** 10.1038/s41598-022-22644-9

**Published:** 2022-10-28

**Authors:** Bhuvaneshwari Shetty, Roshan Fernandes, Anisha P. Rodrigues, Rajeswari Chengoden, Sweta Bhattacharya, Kuruva Lakshmanna

**Affiliations:** 1 Department of Computer Science and Engineering, Government Polytechnic for Women, Mangaluru, 575008 India; 2 Department of Computer Science and Engineering, NMAM Institute of Technology-Affiliated to NITTE (Deemed to be University), Udupi, 574110 India; 3 School of Information Technology and Engineering, VIT, Vellore, 632014 India

**Keywords:** Skin diseases, Computational biology and bioinformatics

## Abstract

Detecting dangerous illnesses connected to the skin organ, particularly malignancy, requires the identification of pigmented skin lesions. Image detection techniques and computer classification capabilities can boost skin cancer detection accuracy. The dataset used for this research work is based on the HAM10000 dataset which consists of 10015 images. The proposed work has chosen a subset of the dataset and performed augmentation. A model with data augmentation tends to learn more distinguishing characteristics and features rather than a model without data augmentation. Involving data augmentation can improve the accuracy of the model. But that model cannot give significant results with the testing data until it is robust. The k-fold cross-validation technique makes the model robust which has been implemented in the proposed work. We have analyzed the classification accuracy of the Machine Learning algorithms and Convolutional Neural Network models. We have concluded that Convolutional Neural Network provides better accuracy compared to other machine learning algorithms implemented in the proposed work. In the proposed system, as the highest, we obtained an accuracy of 95.18% with the CNN model. The proposed work helps early identification of seven classes of skin disease and can be validated and treated appropriately by medical practitioners.

## Introduction

A skin lesion is a growth or appearance of the skin that is abnormal concerning the surrounding skin. Primary and secondary skin lesions are the two types of skin lesions. Primary skin lesions are abnormal skin conditions that can develop over time or be present at birth. Secondary skin lesions can develop from primary skin lesions that have been exacerbated or altered. When a mole is scraped until it bleeds, the crust that forms, as a result, develops a secondary skin lesion^1^. Dermatologists propose one of three treatments for afflicted skin, depending on the type of lesion: home care, medicines, or surgery. Regardless of ways innocent they appear; a few sorts of skin lesions may be pretty risky to the patients, since they will indicate the presence of malignancy and require surgical removal. Melanoma is the most dangerous type of skin cancer; as soon as it has spread, it’s deadly, however, it is treatable in its early stages. As a result, a precise diagnosis of skin patches is essential to protect patients’ growths and ensure that they receive timely treatment^2^.

Machine Learning methods could be used to automate the analysis, resulting in a system and framework in the medical field that would aid in providing contextual relevance, improving clinical reliability, assisting physicians in communicating objectively, reducing errors related to human fatigue, lowering mortality rates, lowering medical costs, and more easily identifying diseases. A machine learning method that can categorize both malignant and benign pigmented skin lesions is a step toward achieving these goals^3^. In the proposed work, Convolutional Neural Networks (CNN) and Machine Learning algorithms are used to accurately classify pigmented skin lesions in dermoscopic images to detect malignant skin lesions as early as feasible.

The HAM10000 dataset which consists of 10015 images has been used in the proposed work.The HAM10000 dataset is a vast collection of dermoscopic images of pigmented skin lesions which are very common from multiple sources^4^. Datasets with significant class imbalances are fairly common in the medical industry. It is the same with this data set. In the proposed work, it proved to be a significant challenge. The dataset images have a resolution of 600 × 450 pixels and are saved as JPEG formats. They are manually cropped and cantered around the lesion, as well as modified for visual contrast and color reproduction, at first. Each image and patient had seven features, namely, age of the patient, sex of the patient, lesion id which is a unique identifier for a particular type of lesion, image id which is a unique identification number for an image, dx type for technical validation, Skin lesion’s geographical location, and a diagnostic skin lesion category which is a classification of skin lesions that can be used to diagnose a condition.

The patients were mostly between the ages of 35 and 70. The ground truth of the data set was represented by the technical validation field category, which revealed how the skin lesion diagnosis was made. Ground truths were divided into four categories by the researchers, namely, Histopathology, Confocal, Follow-up, and Consensus. In the Histopathology category, dermatopathologists diagnosed excised lesions histopathologically. All images were manually evaluated with the relevant histopathologic diagnosis and confirmed for plausibility by the researchers. In the Confocal category, the reflectance confocal microscopy method is used that provides near-cellular resolution, and it was used to confirm the presence of some benign keratoses on the face. In the Follow-up category, the researchers recognized images as proof of biological benignity, if nevi examined with digital dermoscopy confirmed no modifications during 3 follow-up visits or 1.5 years. The consensus category consists of normal benign instances with no follow-up or histology, as well as examples in which two experts have given the same unequivocal benign diagnosis.

**Figure 1:**
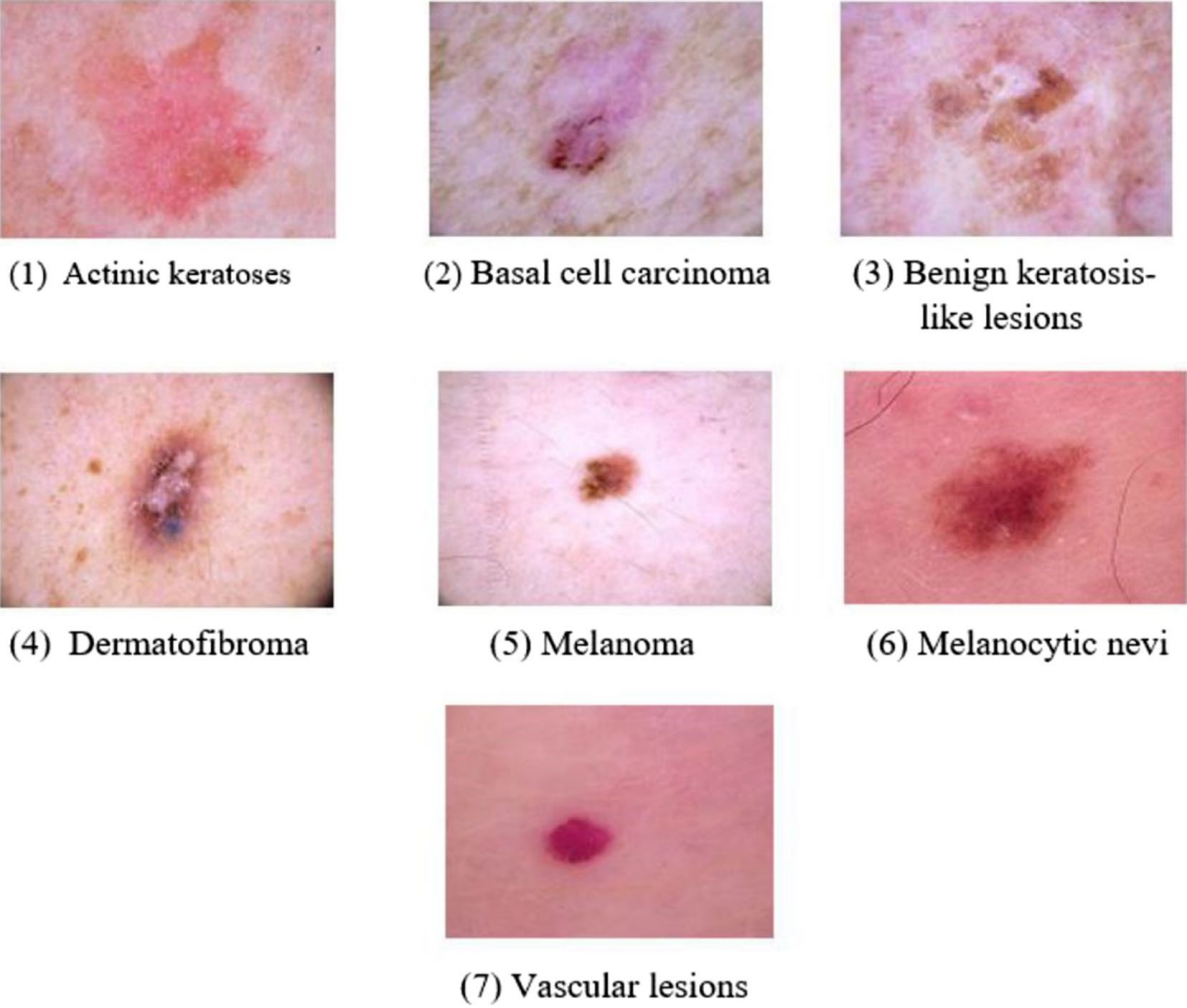
Sample images for the seven skin lesion categories from HAM10000 dataset.

Histopathology was used to diagnose more than half of the skin lesions. The back, lower limbs, and trunk are all significantly impacted skin cancer locations, as demonstrated in the data set’s localization distribution. In terms of diagnostic skin lesion categories, the data set included seven different classes. Figure 1 depicts a selection of sample images from the HAM10000 dataset for each of the classes. The following are the seven categories:

1. Actinic Keratoses [akiec]: Types of squamous cell carcinoma that are noninvasive and can be treated locally without surgery (327 images are available in the data set).
2. Basal Cell Carcinoma [bcc]: A type of epithelial skin cancer that seldom spreads but, if left untreated, can be fatal. (514 images are available in the data set).
3. Benign Keratosis-like Lesions [bkl]: Seborrheic keratoses, lichen-planus like keratoses, and solar lentigo, correlate to a seborrheic keratosis or a sun lentigo with regression and inflammation, are all examples of “benign keratosis” (1099 images are available in the data set).
4. Dermatofibroma [df]: Skin lesions that are either benign growth or an inflammatory response to minor trauma (115 images are available in the data set).
5. Melanoma [mel]: Melanoma is a cancerous tumour that develops from melanocytes and can take many different forms. If caught early enough, it can be treated with a basic surgical procedure (1113 images are available in the data set).
6. Melanocytic Nevi [nv]: Skin lesions are benign neoplasms of melanocytes and appear in a variety of shapes and sizes. From a dermatoscopic standpoint, the variants may differ dramatically (6705 images are available in the data set).
7. Vascular Lesions [vasc]: Cherry angiomas, angiokeratomas, and pyogenic granulomas are examples of benign or malignant angiomas. (142 images are available in the data set).

The proposed work is organized as follows: Section “Literature survey” contains information on similar research in this field. Section “Proposed methodology” details the methodology performed on the data. Section “Results and discussion” discusses all of the model’s performances; Section “Conclusion and future work” contains the conclusions derived from the research and the future work and next steps for this project.

## Literature survey

Dhivyaa et al.^1^ have proposed a model that can produce feature maps of high resolution that can be used to assist in the preservation of the image’s spatial information. On two separate datasets, the authors proposed Random Forest and Decision Tree algorithms for skin class categorization. Polat, Kemal, and Kaan Onur Koc^5^ have proposed a system that uses no filtering and feature extraction. Authors claim to have obtained very encouraging results in the identification of skin lesions. Kumar et al.^6^ have proposed a system with RGB color-space, GLCM, and Local Binary Pattern (LBP) methods for pre-processing and image segmentation that uses fuzzy-c clustering and obtained encouraging results in the identification of skin lesions. Adegun and Viriri^7^ have proposed a system with an encoder-decoder Conditional Random Field (CRF) module for contour refining and lesion boundary localization that uses a linear combination of Gaussian kernels for paired edge potentials. A Fully Convolutional Network (FCN) with hyper-parameters tuning gave good results.

Data augmentation strategies have been proposed by Srinivasu et al.^8^ to balance various forms of lesions to the same range of images. The proposed model, which is predicated on the LSTM and MobileNet V2 approaches, was found to be effective in classifying and detecting skin diseases with little effort and computational resources. When using CNN transfer learning, Mahbod et al.^9^ confirmed that image size affects skin lesion categorization performance. They also showed that image cropping outperforms image resizing in terms of performance. Finally, the best classification performance is demonstrated using a simple ensembling strategy that merges the findings from images clipped at six scales and three fine-tuned CNNs. Zhang et al.^10^ presented an efficiency result of CNN that was optimized using an upgraded version of the whale optimization technique. This technique is used to find the best weights and biases in the network to reduce the difference between the network output and the desired output.

Hameed et al.^11^ proposed a Multi-Class Multilevel (MCML) classification technique inspired by the “divide and conquer” strategy. The proposed classification algorithm combines machine learning and deep learning methods. Hasan et al.^12^ proposed the DSNet, an automatic semantic segmentation network for skin lesions. To reduce the number of parameters and make the network lighter, they used a separable depth-wise convolution. Hosny et al.^13^ used pre-trained AlexNet with transfer learning. As initial values, the parameters from the original model were used. and the weights of the last three replaced layers were randomly initialized. ISIC 2018, the most recent public dataset, was used to test the suggested technique. Chatterjee et al.^14^ used SVM with RBF to identify three lesions by extracting form, fractal dimension, texture, and color variables using fractal-based regional texture analysis (FRTA). Pereira et al.^15^ proposed integrating gradients with the local binary patterns (LBP) technique to increase the performance of skin lesion classification algorithms and to further exploit the border-line properties of the lesion segmentation mask.

A kernel sparse coding approach for both segmentation and classification of skin lesions^16^. A skin lesion detection system was proposed by Garcia-Arroyo et al.^17^ with fuzzy histogram thresholding, lesions were segmented. Using the ABCD rule, Zaqout^18^ has developed a model capable of partitioning and classifying skin pictures. In the majority of these efforts, the ABCD rule is followed, which includes image pre-processing, segmentation to locate the lesion, feature extraction, and classification. TDS is a dermoscopy score that assists in the diagnosis of the lesion condition. Khan et al.^19^ have proposed a system that uses the deep learning model MASK-RCNN for segmentation and pre-trained DenseNet for classification. Using YOLOv5, Shelatkar et al.^20^ describe a deep learning-based method for classifying and identifying brain tumours. To extract the features, a transfer learning concept is used which is then exposed to selection and classification stages. Khan et al.^21^ developed an automated method for skin lesion categorization that used pre-trained RESNET-50 and RESNET-101 deep neural network (DCNN) with transfer learning was employed for feature extraction and optimal feature selection based on the kurtosis-controlled principle component (KcPCA). The information was then combined and the best features were chosen, which were then fed into a supervised learning algorithm—SVM of kernel function radial basis function (RBF) for classification.

Khan et al.^22^ developed a segmentation and classification framework based on deep learning. For skin lesion segmentation, a MASK R-CNN-based architecture with a Resnet50 feature pyramid network (FPN) is used. The final mask is then generated by mapping connected layer-based features. A 24-layer convolutional neural network architecture is built during the classification phase, with activation based on the display of higher characteristics. Finally, the best CNN features are delivered to softmax classifiers for final classification. Harris Hawks Optimization with Deep Learning Model for Detection of Diabetic Retinopathy was proposed by Gundluru et al.^23^. Tajeddin et al.^24^ used highly discriminative characteristics to classify skin melanoma. For lesion segmentation, the authors started with contour propagation. To extract features, lesions were mapped in log-polar space using Daugman’s transformation based on the peripheral area. Finally, the various classifiers used in the proposed work were evaluated.

To construct a dermoscopic skin image recognition system, Yu et al.^25^ recommended CNN and the local descriptor encoding approach. To extract skin lesion features from images, the authors utilized ResNet101 and ResNet50. Using a Fisher vector (FV) and the collected ResNet features, a global image representation was generated. Finally, a Chi-squared kernel was applied in an SVM for classification. Melanoma was categorized into three groups based on the thickness of the lesion^26^. The researchers utilized two categorization schemata: one classified lesion as thin or thick, and the other separated them into thin, moderate, and thick categories. To categorize the lesion data, a combination of ANN and logistic regression algorithms is used. Using a cloud computing system, Rajput et al.^27^ suggested diabetes diagnosis to Indians living in rural locations. To construct a breast cancer detection system, Abbas et al.^2^ proposed the Extremely Randomized Tree and Whale Optimization Algorithm (WOA) to present a unique approach called BCD-WERT for efficient feature selection and classification. To detect ships from satellite imaginary, deep CNN with YOLOv3 for object detection with SHA-256 hashing for security to the detected images^3^. For the purpose of diagnosing heart disease, Reddy et al.^28^ suggested a hybrid genetic algorithm and a fuzzy logic classifier.

To conclude, many researchers have contributed to classifying the skin lesion categories using distinct machine learning and deep learning approaches. Also, they have worked on a variety of data sets. The proposed work mainly aims at classifying the skin lesion categorization on the HAM10000 dataset, which is used by a few researchers. The proposed work concentrates on categorizing the skin lesion into seven classes using different machine learning and Convolutional Neural Network technique and obtained comparatively better results.

### The main contributions of the proposed work

 The HAM10000 dataset images are highly unbalanced. For instance, in the dataset, we observe that Dermatofibroma (df) skin lesion class has a count of 115 images which is the smallest size. The advantage of the proposed work is that we developed a model which is computationally much efficient as compared to existing work as they have considered the entire dataset. This is because a majority of the existing work has considered the entire unbalanced dataset and then they have augmented it to make sure the dataset is balanced. Here they have considered the small class training set (For instance Dermatofibroma (df) skin lesion class) which is augmented around 50 times, which we have avoided in our proposed work. The other contributions include:

- Resizing the medical dermoscopic lesion images to reduce memory consumption and improve latency.
- Data augmentation to overcome the limited number of images and reduce overfitting.
- Global feature descriptors allow the system to extract skin lesion features efficiently even with a little training dataset.
- The proposed customized convolution neural network has hyperparameters to train the model and also, the softmax function is used at the end of the fully connected layer of the CNN. Hence the proposed CNN model works well with binary and multi-class detection.
- To make the model more trustworthy, quicker, and error-free, different evaluation metrics, and 10-fold cross-validation are applied.
- Pipeline for developing a support tool for skin lesion medical practitioners.

## Proposed methodology

**Figure 2:**
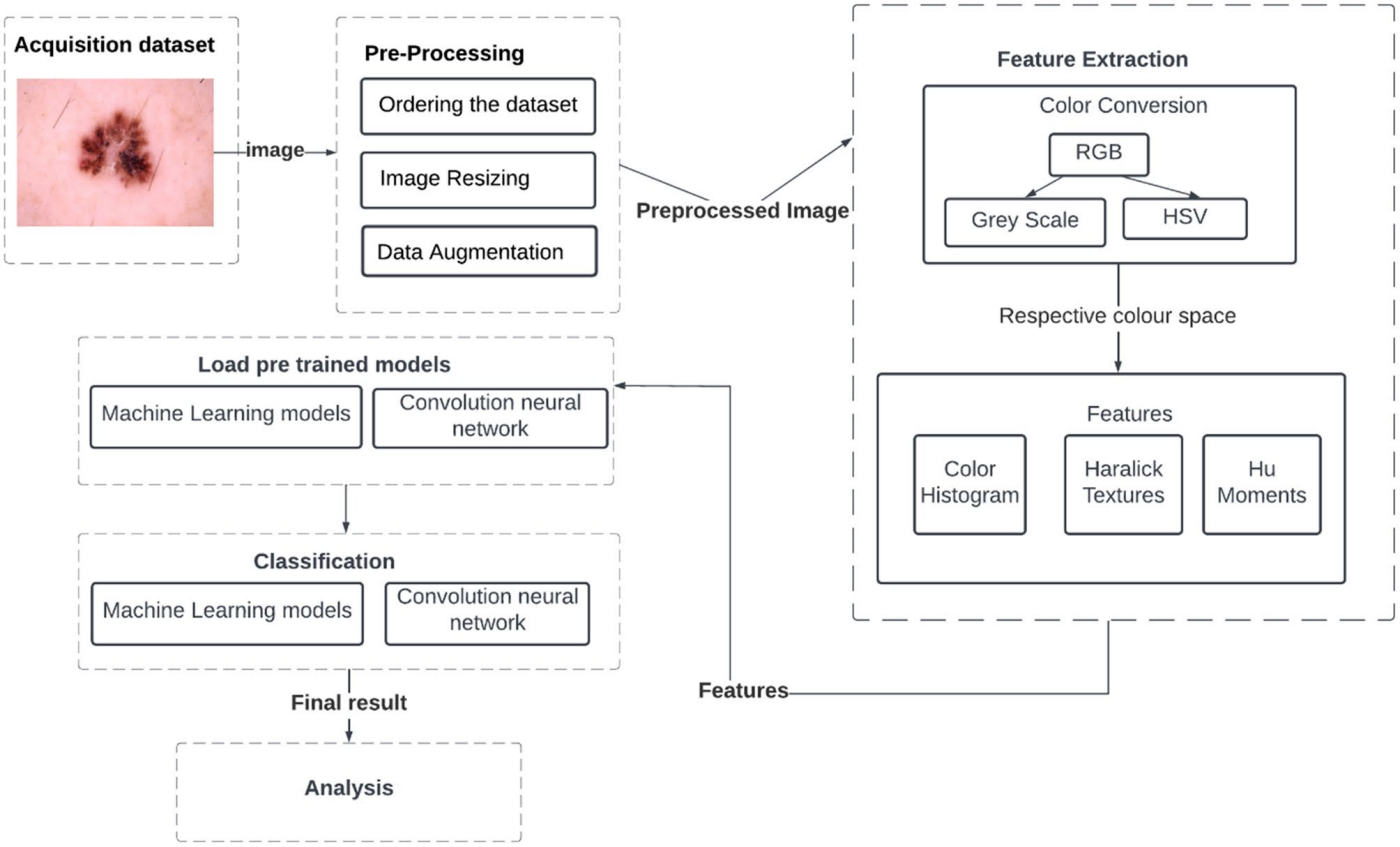
Flowchart of the ML Model.

We developed a fully automated approach for detecting and classifying skin lesions using Machine Learning and customized Convolutional Neural Networks. The proposed work concentrated on pre-processing and classification. The standard HAM10000 dataset is used in the proposed work which contains 10015 skin lesion images divided into seven categories. The steps involved in the proposed work is depicted in Fig. 2.

### Image pre-processing

The proposed work applied the following image pre-processing steps.

#### Step 1 - ordering the dataset

As the dataset images are out of order, sorting each image within each folder by the seven diseases is the necessary step. ’Image id’ and ’dx’ were the most crucial parameters for arranging the images in this scenario. In the dataset, we observe that df skin lesion count has a number 115 which is the smallest size. As a result, choosing 100 images per class and using a dataset of 100*7 images to train the model is insufficient to gain better classification accuracy. As a result, more data will be generated, and data augmentation will be used to achieve this task.

#### Step 2 - Image resizing

1. All the images in the folder are resized to 220*220 before processing into different machine learning models.
2. For the customized CNN model, images are scaled to 96 × 96 with a depth of 3 to speed up the process. Then we have converted the images into a NumPy array to get the value of each pixel of the image. Then we normalized the pixel values to a range of 0–1. The LabelBinarizer class allows us to input class labels that are in string form in the dataset, convert those class labels into one-hot encoded vectors, and then convert them back into a human-readable form from the integer class label prediction of Keras CNN.

#### Step 3 - Data augmentation

**Figure 3:**
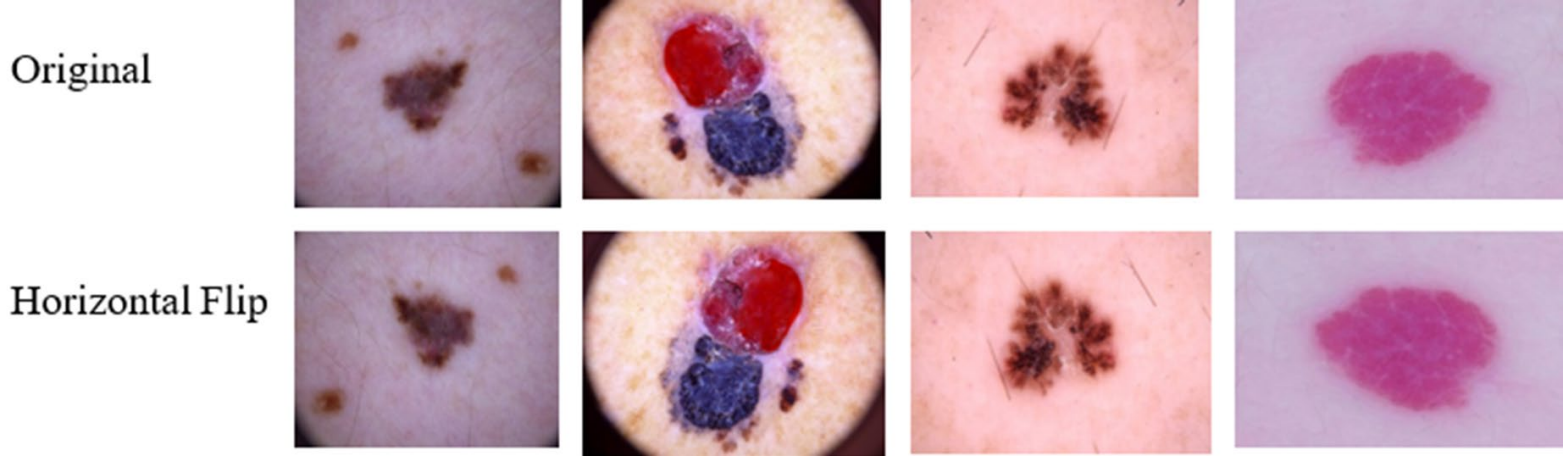
Horizontal Flip augmentation.

1. Data augmentation is a technique for generating new “data”. To train the machine learning models, the proposed method used Horizontal Flip augmentation i.e., shifting all pixels of an image in a horizontal direction. As a result, models with data augmentation are more likely to learn more differentiating characteristic features than models without data augmentation. We have 200 images from each class after augmentation and trained the model with a dataset of 200*7 images. Figure 3 depicts the sample images after Horizontal Flip augmentation.
2. In the CNN model since we are working with a finite number of data points (each class has 200 images), we have applied random transformations (rotations, shearing, etc.) to train. Each epoch had the same number of images as the original images. Overfitting is also avoided by data augmentation.

### Feature extraction

Global Feature Descriptors are used to quantify an image in its entirety. These don’t have the concept of interest points and thus, take the entire image for processing. The color of the skin lesion image is quantified using a Colour Histogram. The shape of the skin lesion is quantified using Hu Moments. The texture of the skin lesion is quantified using a Haralick Texture. These features are chosen because the color, shape, and texture are the only properties that dominate in the lesion zone. The feature extraction experiment works with one image at a time, extracting three global features, concatenating them into a single global feature, and saving it in HDF5 format with its label.

### Data splitting

The OpenCV application was used to validate the machine learning models. The total number of images obtained from the HAM1000 dataset for Machine Learning model training is 700 (100 images from each class), 560 of which images represent 80% for training and 140 images represent 20% for testing. Due to the massive class imbalance revealed by the data set, this was necessary. After augmenting the dataset, 1400 images (200 images from each class) are used for ML model training, with 1120 images accounting for 80% of the training and 280 images accounting for 20% of the testing.

### Image classification

The proposed work used machine learning models and a Convolutional Neural Network model to train and test the image dataset and evaluated the performance using the various parameters, namely, Accuracy, Precision, Recall, and F1-score. The various machine learning models used in the proposed work include Decision Tree (DT), Random Forest (RF), Support Vector Machine (SVM), K-Nearest Neighbor (KNN), Logistic Regression (LR), Gaussian Naïve Bayes (NB), Linear Discriminant Analysis (LDA) at the end, we have compared the various models in terms of the evaluation parameters. The hyperparameters used by the machine learning algorithms are shown in Table 1.

**Table 1:** Machine learning model’s hyperparameter.

Classifier	Hyperparameter value
LR	$random\_state$ = 9
LDA	solver = ’svd’
KNN	$n\_neighbors$ = 5
DT	Estimators = 100
RF	$n\_estimators$ = 200, $random\_state$ = 0
GaussianNB	$var\_smoothing$ = 1e−09
SVM	Kernel = ’linear’, c = 1, $random\_state$ = 0

### Convolutional neural network

**Figure 4:**
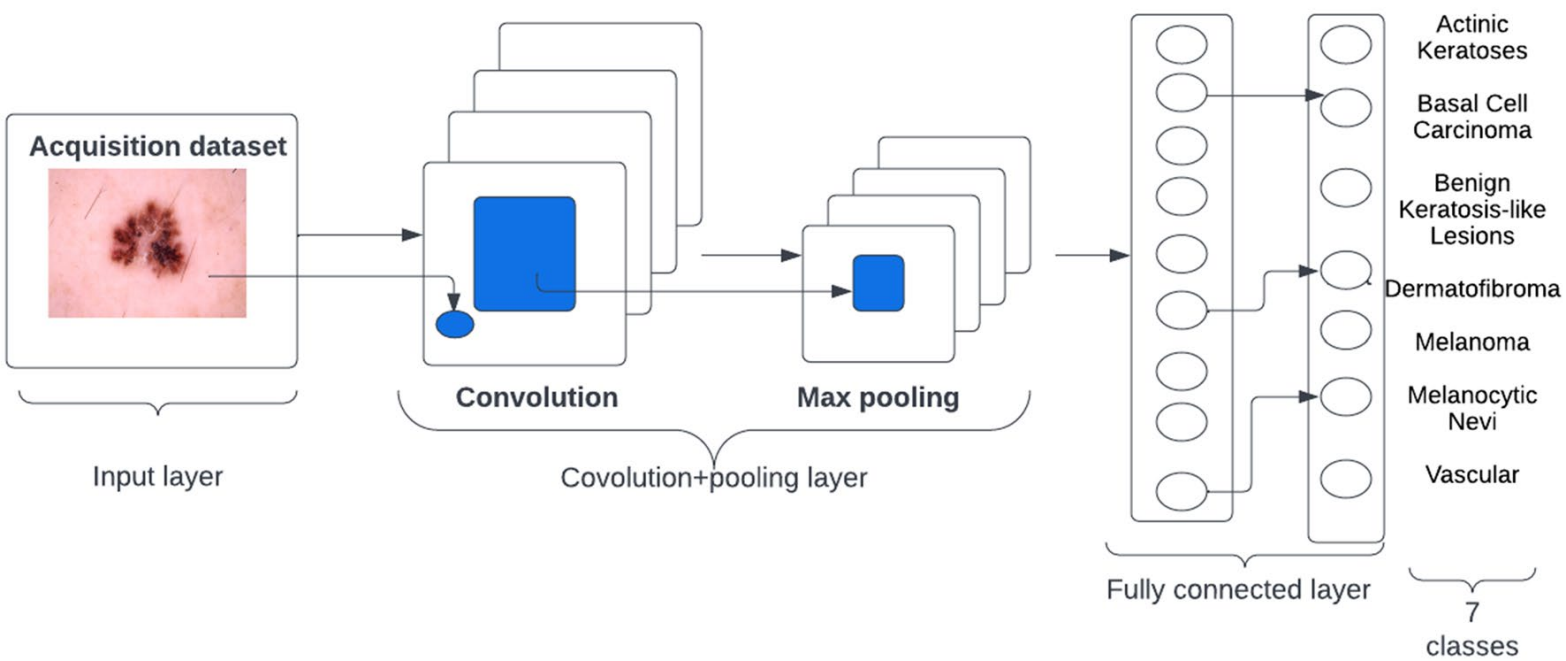
A high-level overview of CNN Architecture.

In contrast to a standard neural net, a CNN learns detailed patterns by applying filters to the raw pixels of an image. To build a CNN, Tensorflow and Keras libraries were used to build and implement the model in Python 3.7.9. A high-level overview of CNN Architecture is shown in Fig. 4. The layers and hyperparameters employed in the network are summarized in Table 2.

**Table 2:** CNN layers and hyperparameters

Layer	Hyperparameters
Conv2D	32 filters, 3 × 3 filter size, ReLU activation, same padding, followed by batch normalization
MaxPool2D	3 × 3 pool size to reduce image spatial dimensions quickly from 96 × 96 to 32 × 32
Dropout (Core Layer)	0.25 Neurons
Conv2D	64 filters, 3 × 3 filter size, ReLU activation, same padding
Conv2D	64 filters, 3 × 3 filter size, ReLU activation, following the same padding, batch normalization is performed
MaxPool2D	2 × 2 pool size
Dropout (Core Layer)	0.25 Neurons
Conv2D	128 filters, 3 × 3 filter, ReLU activation, following the same padding, batch normalization is performed
Conv2D	128 filters, 3 × 3 filter size, ReLU activation, same padding followed by batch normalization
MaxPool2D	2 × 2 pool size
Dropout (Core Layer)	0.25 Neurons
Flatten (Core Layer)	–
Dense	1024 Units, ReLU sctivation, and batch normalization
Dropout (Core Layer)	0.5 Neurons
Dense	7 Units, softmax activation

### Model hyperparameters

To get a better model evaluation, certain common hyperparameter values are used. The hyperparameter values utilized in the CNN model are highlighted in Table 3. The following section explains why the values of the hyperparameters were chosen in the proposed work: Optimizer: Adam is the most widely used optimization method for training deep neural networks today because it is simple to use, computationally efficient, and effective when dealing with enormous amounts of data and parameters. Loss Function: The Multi-Class calculates the loss value using the “categorical cross-entropy” loss function. Epochs: The epoch count is 150. Found that 150 epochs result in a model with low loss and no overfitting to the training set through experimentation (or not overfitted as best as we can). Batch Size: Several early tests with batch sizes of 5, 10, 20, and 40 found that batch size 32 produced the best results. Learning Rate: The rate of learning is initially set to 0.001. The “step” we take along the gradient is controlled by the learning rate. The smaller the value, the smaller the step, and the larger the value, the bigger the step.

**Table 3:** CNN model’s hyperparameters

Hyperparameter	Value
Optimizer	Adam
Loss function	Categorical cross-entropy
Epochs	150
Batch dize	32
Learning rate	0.001–0.00001

## Results and discussion

All of the Machine Learning models and CNN were trained and tested on a Windows 10 computer with an Intel i5 processor and 8GB of RAM. The models were created with Spyder 5 and Python 3.7.9, with Keras, Imutils, and cv2Numpy as dependencies. The accuracy of the machine learning models with two methods involving/not involving augmentation is shown in Table 4.

**Table 4:** Accuracy of the machine learning models.

	LR	LDA	KNN	DT	RF	NB	SVM
Without augmentation for (100*7) images	0.48929	0.35179	0.41786	0.44286	0.58571	0.34107	0.42143
With horizontal flip augmentation for (200*7) images	0.58125	0.57589	0.48393	0.68660	0.87321	0.363393	0.53125

From the Table 4 results, we have observed that the Random Forest Machine algorithm provides better accuracy compared to other machine learning algorithms. The experimental results of CNN and Machine learning outcomes with the use of model accuracy and weighted average of precision, recall and F1-score is shown in Table 5.When using K-fold cross-validation, the accuracy measure is the mean of the accuracies of the K-models, not simply the accuracy of one model.

**Table 5:** Model accuracy, weighted average of precision, recall and F1-score.

Metrics	Model							
	CNN	RF	DT	LR	LDA	SVM	KNN	NB
Accuracy (%)	94	87	68	58	57	53	48	36
Precision (%)	88	94	75	56	62	54	54	49
Recall (%)	85	94	74	55	58	50	50	36
F1-score (%)	86	94	74	55	54	50	50	35

Table 6 gives the customized CNN model’s accuracy and loss for training and testing sets. As seen in this table, the proposed customized CNN model has a performance difference of 9% between the training and testing accuracy. After 150 epochs of training, the model achieved low loss with minimal overfitting. We could also improve our accuracy by adding more training data.

**Table 6:** CNN model training and validation.

CNN model		
	Train	Test
Accuracy (%)	95.18	86.43
Loss	0.1483	0.4690

**Figure 5:**
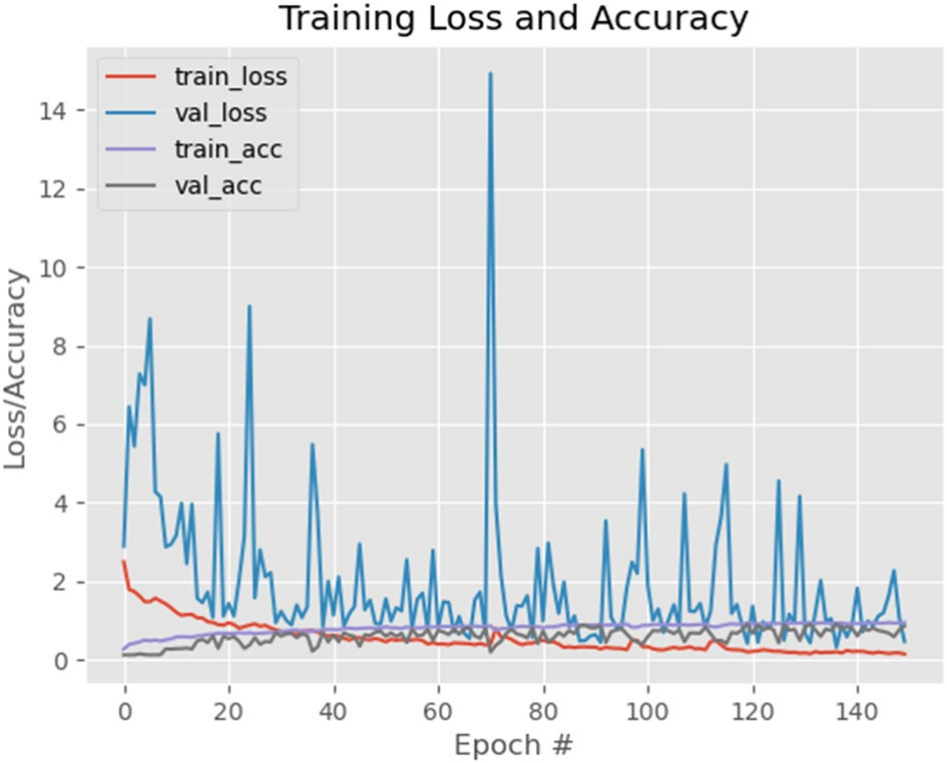
CNN Model’s Accuracy/loss. Model’s performance measure comparison.

Figure 5 depict the CNN model’s training history versus the number of epochs. This is an important visualization to ensure that the model continued to train with each epoch, improving its accuracy and reducing losses while aiming to optimize the objective function. The figures are visible in the code. The testing sets accuracy, recall, precision, and f1-score associated with the model are shown in Table 7.

**Table 7:** Multi-class classification report of the customised CNN model.

	Precision	Recall	F1-score	Support
0-Actinic keratoses	0.89	0.68	0.77	37
1-Basal cell carcinoma	0.55	0.88	0.67	33
2-Benign keratosis-like lesions	0.89	0.94	0.92	35
3-Dermatofibroma	0.88	0.81	0.84	43
4-Melanoma	0.97	0.89	0.93	44
5-Melanocytic nevi	0.93	0.86	0.89	44
6-Vascular lesions	0.95	0.89	0.92	44
Macro avg	0.87	0.85	0.85	280
Weighted avg	0.88	0.85	0.86	280

**Figure 6:**
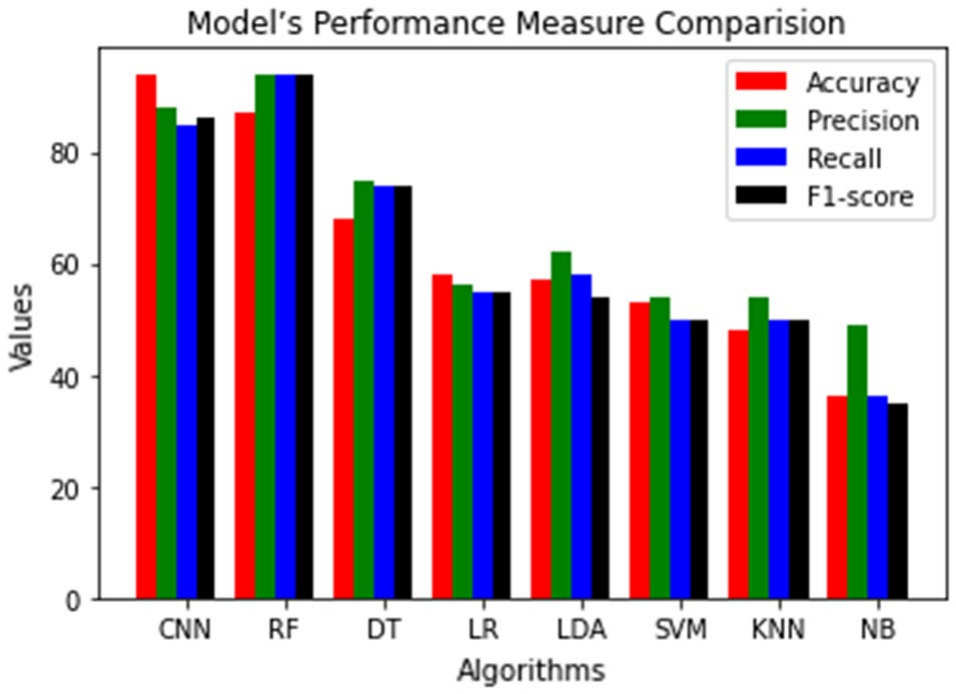
Model’s performance measure comparison.

In Fig. 6 we compare proposed CNN and Machine learning outcomes with the use of model accuracy and weighted average of precision, recall, and f1-score. This is the graphical representation of Table 5. Table 8 gives the comparison of the accuracy obtained in the proposed work with the recent existing work on the HAM10000 dataset. The first row in this table gives the accuracy of the proposed CNN model.

**Table 8:** Comparison of proposed work with recent existing techniques on the HAM10000 dataset.

Existing work versus proposed work	Accuracy (%)
Proposed CNN	95.18
EW-FCM+wide-shufflenet^29^	84.80
Shifted MobileNetV2^30^	81.90
Shifted GoogLeNet^30^	80.50
Shifted 2-Nets^30^	83.20
MobileNetV2-LSTM^8^	85.34
24 layered CNN^22^	86.50
InceptionV3^31^	91.56
ResNetXt101^31^	93.20
InceptionResNetV2^31^	93.20
Xception^31^	91.47
NASNetLarge^31^	91.11
9 layered CNN^32^	80.00
Resnet50, Restnet101+KcPCA+SVM RBF^21^	89.80
Vgg16+googLeNet ensemble^33^	81.50
Modified MobileNet^34^	83.93

We have also implemented Inception V3, a pre-trained model, and compared the accuracy with the proposed work. The InceptionV3 model resulted in an accuracy of 95.14%. Even though there is not much difference in the accuracy, the proposed CNN model has computationally less overhead in terms of total training time as compared to the InceptionV3 model due to more layers in the network.

## Conclusion and future work

The proposed work has applied machine learning and CNN techniques to classify the skin lesion images. The experiments were conducted on the HAM10000 dataset. The machine learning and the customized CNN techniques were evaluated after the experiments based on Accuracy, Precision, Recall, and F1-Score. Before the training/testing phase, the images were pre-processed, then separated into feature and target values, and formed data augmentation. The results show that the customized CNN has obtained an accuracy of 95.18%, which is better than the proposed machine learning algorithms. This suggests that the proposed CNN has a better classification performance for the HAM10000 data set. The proposed work has been compared with the recent existing work on the same data set and proved to obtain better accuracy with minimum loss and errors. As a future work, researchers can improve CNN architecture and implementation by fine-tuning hyper parameters such as the number of layers, type of layers, and hyper parameter values for the layers and can explore other pre-trained CNN models. Researchers can also focus on image segmentation and Skin lesion categorization in real-time with better accuracy and minimum time.

## Data Availability

HAM10000 dataset used in the experiment is available on web publicly at https://www.kaggle.com/datasets/kmader/skin-cancer-mnist-ham10000.
