# Author Correction: Skin lesion classification of dermoscopic images using machine learning and convolutional neural network

**DOI:** 10.1038/s41598-022-26516-0

**Published:** 2022-12-19

**Authors:** Bhuvaneshwari Shetty, Roshan Fernandes, Anisha P. Rodrigues, Rajeswari Chengoden, Sweta Bhattacharya, Kuruva Lakshmanna

**Affiliations:** 1 Department of Computer Science and Engineering, Government Polytechnic for Women, Mangaluru, 575008 India; 2 Department of Computer Science and Engineering, NMAM Institute of Technology-Affiliated to NITTE (Deemed to Be University), Udupi, 574110 India; 3 School of Information Technology and Engineering, VIT, Vellore, 632014 India

Correction to: *Scientific Reports* 10.1038/s41598-022-22644-9, published online 28 October 2022

The Funding section in the original version of this Article was omitted. The Funding section now reads:

“We would like to thank the School of Information Technology and Engineering, Vellore Institute of Technology, Vellore, India − 632014 for paying APC for this study.”

The original Article has been corrected.

